# Functional and esthetic outcomes of redundante rhinoplasty for internal nasal valve dysfunction in Asian patients

**DOI:** 10.1016/j.bjorl.2024.101430

**Published:** 2024-04-03

**Authors:** Hahn Jin Jung, Min Woo Park, Woo Sub Shim, Jee Hye Wee

**Affiliations:** a Chungbuk National University College of Medicine, Chungbuk National University Hospital, Department of Otorhinolaryngology-Head and Neck Surgery, Cheongju, South Korea; b Kangdong Sacred Heart Hospital, Department of Otorhinolaryngology-Head and Neck Surgery, Seoul, South Korea; c Hallym University College of Medicine, Hallym University Sacred Heart Hospital, Department of Otorhinolaryngology-Head and Neck Surgery, Anyang, South Korea

**Keywords:** Rhinoplasty, Nasal obstruction, Nasal septum

## Abstract

**Objective:**

This study aimed to use validated measures to evaluate the functional and esthetic outcomes in patients who underwent functional rhinoplasty for Internal Nasal Valve Dysfunction (INVD) in Korea.

**Methods:**

A retrospective review of consecutive patients who underwent functional rhinoplasty for INVD confirmed by endoscopic findings and the modified Cottle test between 2016 and 2018 was performed. Nasal obstruction was assessed with the Visual Analog Scale (VAS) and nasal obstruction symptom evaluation (NOSE) scale. Acoustic rhinometry was performed pre- and post-operatively. The Minimal Cross-Sectional Area (MCA) of the nose was measured. Objective assessment of the esthetic outcomes was performed with the Objective Rhinoplasty Outcome Score (OROS), which assesses tip rotation, projection, width, dorsal height, width, length, symmetry, and the overall result.

**Results:**

Fifty-seven patients (46 men and 11 women; mean age, 30.5 ± 12.3 years) who underwent functional rhinoplasty were included in this study. The VAS and NOSE scores indicated functional improvement in all cases (all *p* < 0.001). There were no significant between-group differences (VAS score, *p* = 0.274; NOSE score, *p* = 0.952). The objective functional outcomes evaluated using MCA on the concave (*p* = 0.478) and convex (*p* = 0.631) sides did not differ significantly pre- and post-operatively. The subjective evaluation of esthetic satisfaction revealed no between-group difference. Moreover, 31 out of 44 patients (70.5%) with static INVD and nine out of 14 patients (64.3%) with dynamic or combined INVD showed excellent outcomes. Regarding objective esthetic outcomes, scores for the eight factors were >3, and there was no significant difference between the two groups (all *p* > 0.05).

**Conclusions:**

Functional rhinoplasty, including extracorporeal septoplasty and spreader grafting, may be a viable option for correcting INVD with functional and esthetic improvement. Dynamic INVD is less prevalent among Asians, and there was no significant difference in the surgical outcomes compared with those of static INVD.

**Level of Evidence:**

Level 4.

## Introduction

Nasal valve dysfunction is an established cause of nasal obstruction in adults; however, it is frequently overlooked.^1^, ^2^ The nasal valve, first described in 1903,^3^ can be subdivided into external and internal nasal valves. The internal nasal valve is the narrowest portion of the nasal airway, consisting of the caudal portion of the upper lateral cartilage laterally, the dorsal septum medially, and the head of the inferior turbinate inferiorly. Internal Nasal Valve Dysfunction (INVD) can be caused by both static structural abnormalities, such as high septal deviation or hypertrophic turbinate, as well as by a dynamic collapse of the lateral nasal wall during inspiration.^4^, ^5^

Various surgical procedures have been described for the repair of static and dynamic INVD. Spreader grafts and Extracorporeal Septal Reconstruction (ECS) are the most common procedures used for the repair of static INVD.^6^, ^7^ Alar batten, butterfly, and lateral crural strut grafts have been used for strengthening the lateral nasal wall.^8^, ^9^, ^10^ However, studies on the individual effects of each surgical technique are limited. Moreover, there is a lack of research on the surgical techniques used for the repair of static and dynamic valve dysfunction and the extent of functional improvement evaluated using validated measurable objective and subjective outcomes. A systematic review of nasal valve surgery reported that previous studies were frequently focused on technical descriptions of surgical techniques rather than providing evidence of long-term patient benefits.^11^ Moreover, several grafts used for INVD repair may not be esthetically pleasing, indicating the requirement of objective and subjective analyses of the esthetic changes after functional rhinoplasty.

Therefore, this study aimed to assess the functional and esthetic outcomes of functional rhinoplasty for INVD.

## Methods

### Patients

Patients aged ≥ 15-years diagnosed with INVD who underwent functional rhinoplasty for the repair of nasal obstruction persisting for more than 1-year that had not shown satisfactory improvement with medical treatment at the Korea between December 2016 and April 2018 were included in this study. The exclusion criteria included inferior turbinate hypertrophy with allergic rhinitis-associated reversible mucosal edema, revision surgery, a history of nasal trauma, evidence of external nasal valve deformity, and a follow-up period of less than 6-months.

The patients included in this study had INVD with chronic nasal obstruction confirmed by nasal endoscopic examination that was relieved using the Cottle and modified Cottle maneuvers. The Cottle maneuver^12^, ^13^ involved applying gentle lateral digital traction on the cheek adjacent to the nose, whereas the modified Cottle maneuve^14^, ^15^ involved gently supporting the lateral wall cartilage using a cotton tip applicator on each side of the nose. INVD is divided into static and dynamic types. Static INVD is defined as narrowing or stenosis of the angle between the upper lateral cartilage and nasal septum and is caused by severe caudal and/or high septal deviation involving the L-strut. Dynamic INVD is defined as active narrowing of the valve that occurs only with deep nasal inspiration, secondary to a weakness in the integrity of the upper lateral cartilage or nasal side wall.

The Institutional Review Board of the Chungbuk National University Hospital approved the study protocol (no. 2020-06-029) and was conducted in accordance with the principles of the Declaration of Helsinki. Due to the retrospective nature of the study, the need for written informed consent was waived.

### Surgical procedures

All surgeries were performed by an experienced surgeon (W.S.S.), who used anterior rhinoscopy and endoscopy to evaluate the preoperative status of the nasal valve. After inducing general anesthesia, the osseocartilaginous skeleton was exposed, and the septal mucoperichondrial flaps were elevated to perform septoplasty and cartilage graft harvesting. Spreader grafts were fashioned into a rectangular shape on the back table using the harvested septal cartilage. The caudal ends of the Upper Lateral Cartilage (ULC) were separated, and the designed spreader grafts were inserted into the unilateral or bilateral dissected pockets within the internal nasal valve. The grafts were secured to the dorsal septum using 5-0 Nylon, and the angle and cross-sectional area of the internal nasal valve were increased via lateral expansion of the ULC.

A flaring suture is a horizontal mattress suture extending from one ULC to another over the nasal dorsum. Both ULCs flare dorsally once the sutures are tightened, thereby increasing the cross-sectional area and angle of the internal valve. The suture widens the internal valve; however, sidewall collapse is prevented by the suture tension.

Alar batten grafts are cartilaginous grafts comprising of the conchal cartilage that are placed in precise pockets along the point of maximal collapse and its lateral aspect was overlapped with the piriform aperture to adequately support the nasal sidewall and prevent dynamic collapse. The placement of graft was determined via a modified Cottle maneuver which assessed the site of the nasal sidewall collapse.

Butterfly grafts harvested from the conchal cartilage of the ear are structurally supportive onlay grafts. The wedge-shaped carved graft was positioned superficial to the anterior septal angle and caudal edge of the upper lateral cartilage. The caudal end of the graft was positioned within the cephalic margin of the lower lateral cartilage. The graft provides an outwardly spring effect, thereby widening and supporting the upper lateral cartilage, in addition to supporting the lower lateral cartilage. This procedure results in widening of the internal valve angle and an increase in cross-sectional area.

Extracorporeal septoplasty is a surgical technique frequently used at our hospital for the correction of INVD.^16^ Extracorporeal septoplasty involves the removal of the entire septal cartilage, except for the approximately 10 mm of the dorsal stump in place to prevent injury to the keystone. The twisted septal cartilage was cut at its maximum deflection angle, and a neo-L strut was designed subsequently. The neo-L strut was implanted back at its anatomic position and secured to the stump in the dorsum and soft tissues around the anterior nasal spine using 4-0 polydioxanone figure-of-eight sutures.

The remainder of the rhinoplasty was completed subsequently. Additional procedures, such as dorsal augmentation, cartilage grafting, cartilage suture techniques, or osteotomy, were performed, if necessary. Lastly, the incision was closed using 6-0 nylon sutures, and external nasal splints were applied. Post-operative follow-up visits were held at 1-week, 1-month, 3-months, 6-months, 1-year and annually thereafter.

### Evaluation of the subjective and objective outcomes

Information regarding the demographic data, operation time, and postoperative complications was collected via the review of the electronic medical charts. The subjective satisfaction of the patients with the functional and esthetic results was evaluated using a questionnaire that included items on the self-evaluated outcomes of postoperative nasal appearance and obstruction, which were graded as excellent, good, moderate, and poor. Nasal obstruction was graded using a 10-point Visual Analog Scale (VAS), in which 0 indicated the absence of nasal obstruction, and 10 indicated severe obstruction, and a Nasal Obstruction Symptom Evaluation (NOSE) scale,^17^ in which 0 indicated no obstruction, and 4 indicated severe obstruction pre- and post-operatively.

The objective functional outcomes were evaluated using the Minimal Cross-sectional Area (MCA) of the concave and convex sides of the internal nasal valve region using acoustic rhinometry. Mucosal decongestion was induced via the administration of 0.25% phenylephrine spray to minimize the effects of the nasal cycle. Two rhinoplasty surgeons blinded to the purpose of the study compared preoperative photographs with photographs taken at the last follow-up visit to objectively evaluate the esthetic results. The postoperative results were graded using the Objective Rhinoplasty Outcome Score (OROS), a modified version of the rhinoplasty outcome score proposed by Chin and Uppal.^18^ Eight components (tip rotation, projection, width, dorsal height, width, length, symmetry, and overall results) were evaluated on a five-point scale (0 = poor; 1 = no improvement; 2 = moderate; 3 = good; 4 = excellent).

### Statistical analysis

All continuous data are presented as mean ± standard deviation. A non-parametric paired test was performed to compare continuous variables pre- and post-operatively using the Wilcoxon signed-rank test. Student’s *t-*test was used to compare continuous data across the categories. Categorical data are expressed as frequencies and percentages and were compared using the Chi-Square test. Statistical analyses were performed using SPSS software (version 20.0; IBM Corp., Armonk, NY, USA). Statistical significance was assessed by a *p-*value of less than 0.05.

## Results

Table 1 shows the general characteristics of the participants. Fifty-seven patients (46 men and 11 women) underwent functional rhinoplasty for INVD. The mean age was 30.5 years (range, 15–68 years). The mean operation time was 89.5 min. The postoperative mean follow-up period was 10.4 months (range, 6–19 months). Static, dynamic, and combined static and dynamic INVD were observed in 44, two, and 11 patients, respectively. Among the 55 patients with static INVD, 33 (75.0%) underwent spreader grafting, and 29 (65.9%) underwent ECS. Among the 13 patients with dynamic INVD, 6 (46.2%) underwent butterfly grafting, and 4 (30.8%) underwent alar batten grafting. Two patients with only dynamic INVD underwent butterfly and alar batten grafting.

**Table 1:** General characteristics of participants.

	Static (n = 44)	Dynamic (n = 2)	Combined (n = 11)
**Sex**			
Male (n, %)	38 (86.4)	1 (50.0)	7 (63.6)
Female (n, %)	6 (13.6)	1 (50.0)	4 (36.4)
Age (mean, SD)	29.5 (12.4)	42.0 (21.2)	32.5 (10.5)
**Graft**^a^			
Spreader (n, %)	33 (75.0)	0 (0.0)	5 (45.5)
ECS (n, %)	29 (65.9)	0 (0.0)	4 (36.4)
Butterfly (n, %)	0 (0.0)	1 (50.0)	5 (45.5)
Alar batten (n, %)	0 (0.0)	1 (50.0)	3 (27.3)

ECS, Extracorporeal Septal Reconstruction.

a Non-mutually exclusive events.

**Figure 1:**
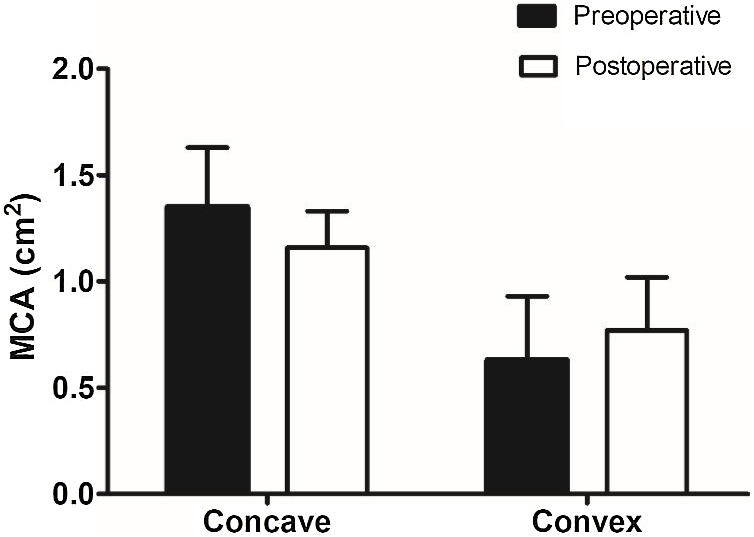
**Objective functional evaluation with acoustic rhinometry.** A decrease in the MCA is observed on the concave side of the nasal cavity, whereas an increase is observed on the convex side; however, the differences are not statistically significant. (MCA, Minimal Cross-sectional Area).

Table 2 presents the pre- and post-operative VAS scores for nasal obstruction intensity and the NOSE scores of the static and dynamic or combined INVD groups. All patients included in this study showed improvements in nasal breathing postoperatively. The paired *t*-test revealed that the VAS (7.9 ± 1.2 preoperatively and 3.0 ± 1.3 postoperatively, *p* < 0.001 in the static group; 8.2 ± 0.9 preoperatively and 3.5 ± 1.2 postoperatively, *p* < 0.001 in the dynamic or combined group) and NOSE (16.6 ± 2.4 preoperatively and 7.8 ± 1.7 postoperatively, *p* < 0.001 in the static group; 17.0 ± 1.0 preoperatively and 7.4 ± 1.5 postoperatively, *p* < 0.001 in the dynamic or combined group) scores were significantly improved in all groups. There were no significant between-group differences (VAS score, *p* = 0.274; NOSE scale score, *p* = 0.952). Fig. 1 presents the results of the objective functional evaluations of both groups. The MCA decreased from 1.35 cm^2^ to 1.16 cm^2^ on the concave side of the nasal cavity, whereas it increased from 0.63 cm^2^ to 0.77 cm^2^ on the convex side; however, the differences were not statistically significant (*p* = 0.631 and *p* = 0.478, respectively) (Fig. 1).

**Table 2:** Preoperative and postoperative comparison of subjective functional outcomes between static and dynamic/combined internal nasal valve dysfunction.

	VAS (mean, SD)		*p*-value^b^	NOSE (mean, SD)		*p*-value^b^
	Pre	Post		Pre	Post	
**Static**	7.9 (1.2)	3.0 (1.3)^a^	0.274	16.6 (2.4)	7.8 (1.7)^a^	0.952
**Dynamic or Combined**	8.2 (0.9)	3.5 (1.2)^a^		17.0 (1.0)	7.4 (1.5)^a^	

VAS, Visual Analog Scale; NOSE, Nasal Obstruction Symptom Evaluation.

a Paired *t*-test, significance at *p* < 0.001.

b Repeated-measure analysis of variance.

**Figure 2:**
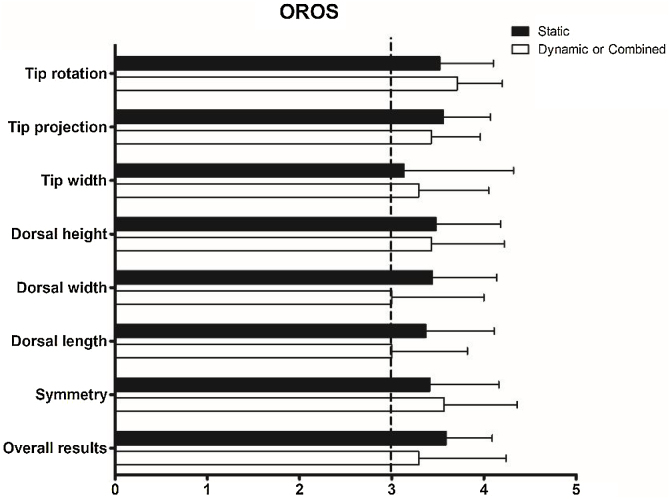
**Objective evaluations of the aesthetic outcomes with OROS.** The scores for the eight factors are >3 in both groups. No significant difference is observed between each score of the two groups. (OROS, Objective Rhinoplasty Outcome Score).

Subjective satisfaction with nasal appearance was determined to be excellent in 31 cases (70.5%) and good in 12 cases (27.3%) in the static INVD group, and excellent in 9 cases (69.2%) and good in 4 cases (30.8%) in the dynamic or combined INVD group. Fig. 2 presents the results of the objective evaluations of the esthetic outcomes. The scores for the eight factors were >3 in both groups. There were no significant differences between the groups in terms of tip rotation (*p* = 0.383), tip projection (*p* = 0.585), tip width (*p* = 0.678), dorsal height (*p* = 0.875), dorsal width (*p* = 0.302), dorsal length (*p* = 0.305), symmetry (*p* = 0.631), or overall results (*p* = 0.437).

Complications, such as graft infection, extrusion, fullness, or visibility, did not occur in any patient.

## Discussion

We evaluated the functional and esthetic outcomes of functional rhinoplasty for INVD in patients of Asian descent and compared the surgical outcomes based on the presence of dynamic INVD. Although multiple surgical procedures were used in functional rhinoplasty for INVD, both subjective and objective evaluations revealed significant improvements in nasal obstruction and esthetic results, regardless of dynamic INVD.

In this study, spreader grafting (69.1%) was primarily used for functional rhinoplasty in patients with static INVD. Spreader grafts, which were first described by Sheen,^6^ are the gold standard technique used for repairing INVD. Spreader grafts provide increased tension to open the nasal valve angle and maintain a straightened position of the septal cartilage.^19^ Previous studies have shown objective functional improvements in nasal obstruction and/or acoustic rhinometry measurements using the VAS scores following functional rhinoplasty with a spreader graft in patients with INVD.^20^, ^21^, ^22^ However, spreader graft placement can result in the widening of the middle-third of the nasal dorsum with less-defined esthetic lines. Few studies have reported the esthetic results of rhinoplasty using spreader grafts, and some studies only subjectively evaluated esthetic satisfaction.^23^ The present study evaluated the objective esthetic results using OROS and revealed the scores for the overall results and dorsal width were 3.5 and 3.4 (between good and excellent), respectively.

ECS, which was initially described in 1952 by King and Ashley, is a useful technique for the correction of severely deviated nasal septum.^24^ ECS can be used in cases where anterior septal deviation is the main cause of nasal obstruction, leading to INVD. ECS achieves esthetic and nasal functional improvements as the surgeon can control the tip position of the neo-L-strut.^16^ A recent systematic review and meta-analyses of the functional and esthetic outcomes of ECS reported that the 17 included studies did not focus on the treatment of INVD, and only three studies reported the esthetic outcomes.^25^ A previous study demonstrated the functional results of the ECS, focusing on internal nasal valve stabilization;^26^ however, the functional results were based on rhinomanometry and acoustic rhinometry. Recent research has shown that ECS can be functionally and esthetically beneficial for tip projection (nasal tip projection (0.62° to 0.66°, *p* < 0.033), nasofrontal angle (152.3° to 148.1°, *p* < 0.001), and nasolabial angle (88.8° to 92.0°, *p* < 0.001)).^16^ In the present study, ECS with or without the use of a spreader graft was performed in 60.0% of the patients with static INVD, and it was found that the subjective and objective results for nasal obstruction and esthetic outcomes improved significantly.

In the present study, only 22.8% of the patients had dynamic INVD. A previous study reported that a wider internal nasal valve angle (21.6° ± 4.5°) than that in Caucasians (11.4° ± 2.6°) and relatively thick, soft tissue envelopes/skin prevent the collapse of the lateral nasal wall in patients of Asian descent.^27^ Although only a small number of patients with dynamic INVD were included, no significant differences in the surgical outcomes were observed. Butterfly and alar batten grafts were primarily used for the correction of dynamic INVD. The use of butterfly grafts, first introduced by Clark and Cook^28^ as an alternative to the spreader graft, has gained popularity for the correction of dynamic INVD during primary and secondary rhinoplasty.^29^, ^30^, ^31^ However, butterfly graft is associated with poor cosmetic result,^32^ as there are concerns regarding the placement of a bulky graft over the dorsum, which may result in middle-third widening with visible graft. However, several refined techniques, such as beveling the edges of the graft, shaving the dorsum of the caudal septum, or placing crushed cartilage for camouflage, have circumvented this complication.^32^ In this study, the patients in the dynamic INVD group who underwent butterfly grafting showed good esthetic outcomes. Alar batten grafting was first introduced by Toriumi et al.^33^ as an effective technique for the correction of dynamic INVD. A previous study reported that alar batten grafting alone provided structural integrity to the INV and produced good outcomes for nasal obstruction and quality of life.^2^

The present study had some limitations. First, multiple surgical techniques were performed concurrently. Due to the characteristics of rhinoplasty procedures, which involve a combination of several techniques, it is not possible to conduct a randomized or controlled study. The main objective of this study was to demonstrate the functional and esthetic outcomes of functional rhinoplasty for INVD. Second, an endoscopic examination was performed instead of comparing the angles of the internal nasal valve in the present study. A previous review stated that the nasal valve area can be better visualized with an endoscope without manipulating the lateral nasal wall and caudal upper lateral cartilage.^34^ Third, patients with external nasal valve dysfunction were excluded from the present study. Except for a few congenital cases, external nasal valve dysfunction is mainly observed in patients undergoing revision rhinoplasty. These cases were excluded from the present study due to the small sample size. Lastly, there is no consensus on the most reliable objective evaluation for the assessment of nasal obstruction. A previous study^20^ used a validated patient self-evaluation module (NOSE score)^17^ in addition to objective acoustic rhinometry. Similarly, the present study also used the VAS, NOSE scores, and acoustic rhinometry. Although the improvement in MCA did not achieve statistical significance, the improvement of the nasal obstruction appears to have been successfully demonstrated when considering both the objective and subjective evaluation methods. Furthermore, a previous review^35^ indicated that uncertainty regarding the correlation between outcomes measured with acoustic rhinometry and an individual's subjective sensation of nasal patency and no basis was identified to prioritize the value of objective outcomes over subjective patency symptoms in evaluating therapeutic interventions.

Nevertheless, the present study is valuable in that only patients who underwent primary functional rhinoplasty for INVD were included. Many surgical techniques described for nasal valve reconstruction have focused on secondary nasal surgery to improve nasal function following rhinoplasty.^6^, ^28^, ^36^ Owing to a better understanding of the nasal valve, recent improvements in surgical techniques, and thorough preoperative evaluation, INVD can often be identified in patients without a history of rhinoplasty who present with nasal obstruction. In addition, both subjective and objective evaluations of the functional and esthetic outcomes were performed in the present study.

## Conclusion

Functional rhinoplasty, including ECS and spreader grafting, may be a viable option for the repair of INVD with functional and esthetic improvements. Dynamic INVD is less prevalent in patients of Asian descent, and there was no significant difference in the surgical outcomes compared with those of static INVD.

## Funding

None.

## Conflicts of interest

The authors declare no conflicts of interest.
